# Pancreatic cancer ascites xenograft–an expeditious model mirroring advanced therapeutic resistant disease

**DOI:** 10.18632/oncotarget.17253

**Published:** 2017-04-19

**Authors:** Talia Golan, Chani Stossel, Michael Schvimer, Dikla Atias, Sharon Halperin, Ella Buzhor, Maria Raitses-Gurevich, Keren Cohen, Sara Pri-Chen, Julie Wilson, Robert E. Denroche, Ilinca Lungu, John M.S. Bartlett, Faridah Mbabaali, Yosef Yarden, Nishanth Belugali Nataraj, Steven Gallinger, Raanan Berger

**Affiliations:** ^1^ Pancreatic Cancer Translational Research Laboratory, Oncology Institute, Sheba Medical Center, Tel Hashomer, Israel; ^2^ Sackler Faculty of Medicine, Tel Aviv University, Tel Aviv, Israel; ^3^ Pathology Department, Sheba Medical Center, Tel Hashomer, Israel; ^4^ Microsurgery Laboratory, Eye Institute, Sheba Medical Center, Tel Hashomer, Israel; ^5^ Ontario Institute for Cancer Research, Toronto, Canada; ^6^ Department of Surgery, University Health Network, Toronto, Canada; ^7^ Department of Biological Regulation, Weizmann Institute of Science, Rehovot, Israel; ^8^ Oncology Institute, Sheba Medical Center, Tel Hashomer, Israel

**Keywords:** pancreatic ductal adenocarcinoma (PDAC), metastatic, ascites, whole genome sequencing (WGS), patient derived xenograft (PDX)

## Abstract

Pancreatic ductal adenocarcinoma has limited treatment options. There is an urgent need for developing appropriate pre-clinical models recapitulating metastatic disease, the most common clinical scenario at presentation. Ascites accumulation occurs in up to 20–30% of patients with pancreatic cancer; this milieu represents a highly cellular research resource of metastatic peritoneal spread. In this study, we utilized pancreatic ascites/pleural effusion cancer cells to establish patient derived xenografts.

Ascites/pleural effusion-patient derived xenografts were established from twelve independent cases. Xenografts were serially passed in nude mice and tissue bio-specimen banking has been established. Histopathology of emergent tumors demonstrates poorly to moderately differentiated, glandular and mucin producing tumors, mirroring morphology of primary pancreatic cancer tumors. Whole genome sequencing of six patient derived xenografts samples demonstrates common mutations and structural variations similar to those reported in primary pancreatic cancer. Xenograft tumors were dissociated to single-cells and *in-vitro* drug sensitivity screen assays demonstrated chemo-resistance, correlating with patient clinical scenarios, thus serving as a platform for clinically relevant translational research.

Therefore, establishment of this novel ascites/pleural effusion patient derived xenograft model, with extensive histopathology and genomic characterization, opens an opportunity for the study of advanced aggressive pancreatic cancer. Characterization of metastatic disease and mechanisms of resistance to therapeutics may lead to the development of novel drug combinations.

## INTRODUCTION

Pancreatic ductal adenocarcinoma (PDAC) is one of the most lethal malignancies with limited treatment options. Drug combinations including FOLFIRINOX or gemcitabine plus nab-paclitaxel have improved progression free survival and overall survival [1, 2], yet the 5-year survival rate remains dismal ~6% [3]. To date, surgical resection is the only potential cure; however, only 15–20% of patients are eligible for surgery, and most still die within two years of surgery due to recurrence of disease [4]. Most patients are diagnosed with metastatic disease and treatment options are limited. Molecular phenotypes are emerging in PDAC and the therapeutic relevance of these phenotypes at advanced disease needs to be thoroughly investigated. Recent classification of primary PDAC into subtypes from whole genome sequencing (WGS) data has been performed [5, 6]. The most clinically relevant subtype has genomic instability enriched with a mutational signature of DNA damage repair deficiency [7].

Translational research requires well-validated pre-clinical models that reflect the pathological, cellular and molecular properties of human PDAC. Established cell lines undergo selective pressure and genetic drift during decades of *in vitro* passaging [8]. Genetically engineered mice (GEM) with mutations in KRAS and p53 [9], SMAD4 [10] and P16/INK4 [11] have been established, however, these models frequently have incomplete penetrance, long latency periods and are limited to specific, predefined genetic mutations [12].

Models that more accurately reflect tumor heterogeneity and tumor complexity are needed. Hidalgo et al, implanted 94 resected primary PDAC of which 61% successfully engrafted [13]. These patient derived xenografts (PDX) closely recapitulate human tumor histopathology, including stroma formation, and with preserved heterogeneity [14, 15]. PDX have also been established from PDAC fine needle aspirates [16] and liver metastasis core-needle biopsies [17]. These xenografts derived directly from patient samples, without *in vitro* manipulation, provide a more accurate depiction of stage dependent biologic characteristics and may have complimentary value in characterizing advanced PDAC [18–21, 22].

Most common sites for pancreatic metastases include liver, lung and the peritoneum. Approximately one third of patients with PDAC have peritoneal spread of disease, which may lead to ascites accumulation [23, 24] and palliative paracentesis is performed when clinically required. Pleural effusion also occurs in a small percentage of patients. Symptomatic fluid accumulation usually occurs at the clinically treatment resistant stage, late in the course of the disease. Ascites and pleural effusion contains viable tumor cells [25], and therefore it is compelling to develop investigational models based on primary tumor cells obtained from this fluid.

We have previously characterized the utilization of PDAC ascites fluid as a platform for personalized medicine [26]. Herein, we describe the establishment of ascites and pleural effusion – derived PDX and a detailed histopathology and whole-genome sequencing analysis of representative cases.

## RESULTS

### Clinical characterization

Herein we present clinical characteristics of seventeen PDAC patients with malignant ascites or pleural effusion. Median overall survival of all patients was 8.2 months. Mean time from paracentesis to death was 42 days. Clinical data is described in Supplementary Table 1.

### Establishment of ascites and pleural effusion PDAC patient-derived xenograft model

**Figure 1:**
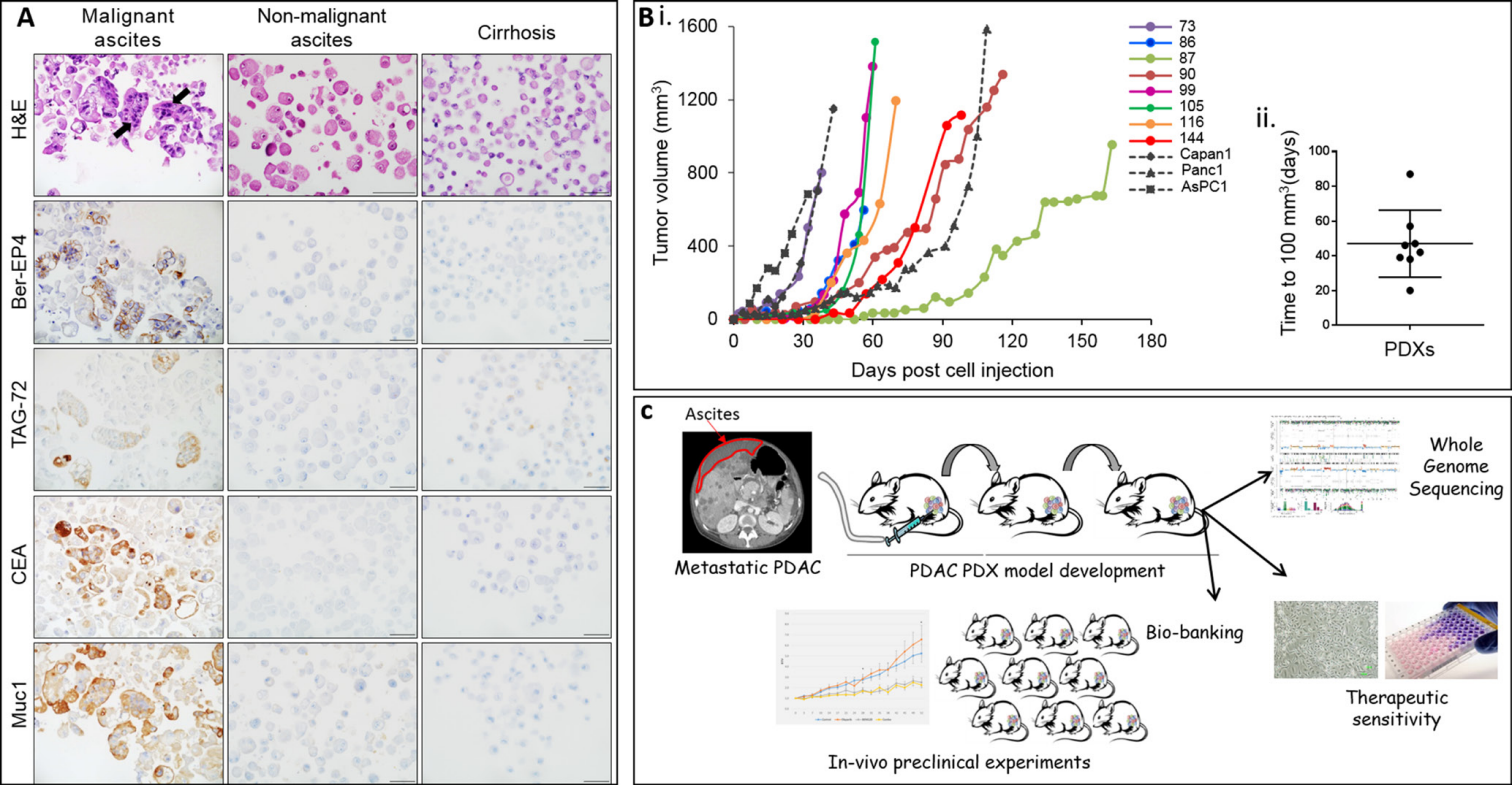
Cytological characterization of ascites cultures and PDX tumor growth kinetics (**A**) H&E and immuno-cytostaining for BerEP4, TAG-73, CEA and Muc1 epithelial markers of a representative malignant PDAC ascites cell block (left column), non-malignant ascites (middle column) and cirrhosis ascites (right column). Magnification ×600. (**B.i**) Tumor growth kinetics of ascites/pleural effusion PDX. Tumor volume as a function of days’ post cell injection of representative F1 generation PDX of each patient (*n* = 1–3). Growth kinetics of established cell lines (AsPC1, Panc1 and Capan1 is represented in grey dotted curves). (**B.ii**) Whisker box plot representing time to tumor palpation (100 mm^3) of PDX. (**C**) Ascites/pleural effusion PDX model illustration.

Malignant ascites and pleural effusion fluid demonstrated tumor cells positive for Muc1, Ber-EP4, TAG-72 and CEA (Figure 1A, arrow) (*n* = 20). Ascites fluid from cirrhosis patients served as control, demonstrating mesothelial cells and lymphocytes. To date, a total of 20 malignant ascites fluids (AF) and pleural effusions (PE) (PE 2/20) from 17 different patients, were subcutaneously injected into athymic nude mice to generate first (F1) generation PDX (*n* = 1 or 2). Successful tumor engraftment was demonstrated in 12/17 patients (70%). Herein we present phenotypic (*n* = 8) and genomic molecular characterization (*n* = 6) of established PDXs.

A minimal detectable tumor (100 mm^3^) was observed 20–87 days after cells injection (mean- 44), and an exponential growth trend was observed thereafter illustrating variation in tumor growth kinetics between PDXs (Figure 1Bi, ii). Xenografts from established pancreatic cell lines (Capan1, AsPC1 and Panc1) were generated for comparison (Figure 1Bi; grey curves). Serial passaging was performed from all xenografts with 100% uptake. A PDX tissue bank for future preclinical experiments was generated. Third- generation PDXs were excited and preserved in Liquid Nitrogen. Representative frozen PDXs were thawed and transplanted to mice with ~90% uptake.

We propose an expeditious unique method for generation of metastatic PDAC PDX model (Figure 1C).

### Histopathologic characterization of PDX

**Figure 2:**
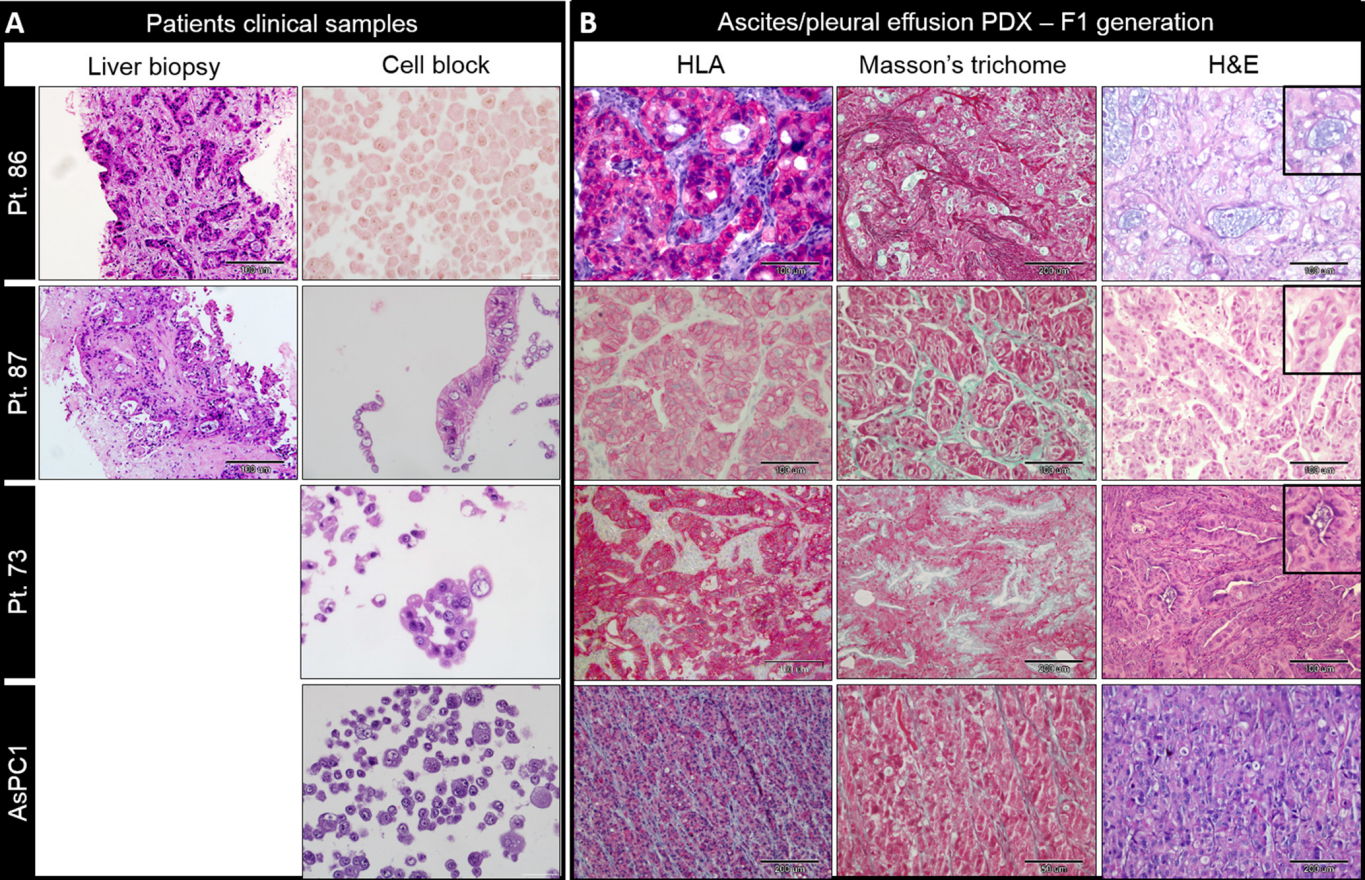
Histopathological characterization of ascites/ pleural effusion PDX (**A**) H&E staining of patient clinical samples; Diagnostic liver biopsy (left column) and ascites cell block (right column) of three representative patients (pt. 73, pt. 86 and pt. 87) and AsPC1 pancreatic cancer cell line; scale bar for liver biopsy – 100 μm; magnification ×400 for ascites cell block. (**B**) HLA-A (left column), Masson's trichome (middle column) and H&E (right column) of PDX tumors. Mucin production (insets). Undifferentiated tumor sheets generated from AsPC1 established cell line (bottom row).

Pancreatic primary tumors and liver metastasis are characterized by desmoplastic stroma containing fibroblasts, endothelial and immune cells. Our PDX tumors were compared to clinical diagnostic tissue when available (Figure 2A). Histologic evaluation of PDX's demonstrated glandular forming moderately to poorly differentiated adenocarcinoma. Mucin production was evident in five PDXs (Figure 2B). In contrast, tumors generated from established pancreatic cell lines demonstrated sheets of undifferentiated tumor cell growth with <5% gland formation. Human leucocyte antigen (HLA) staining was positive in cancer epithelial cells whereas stromal cells remained unstained (Figure 2B).

**Figure 3:**
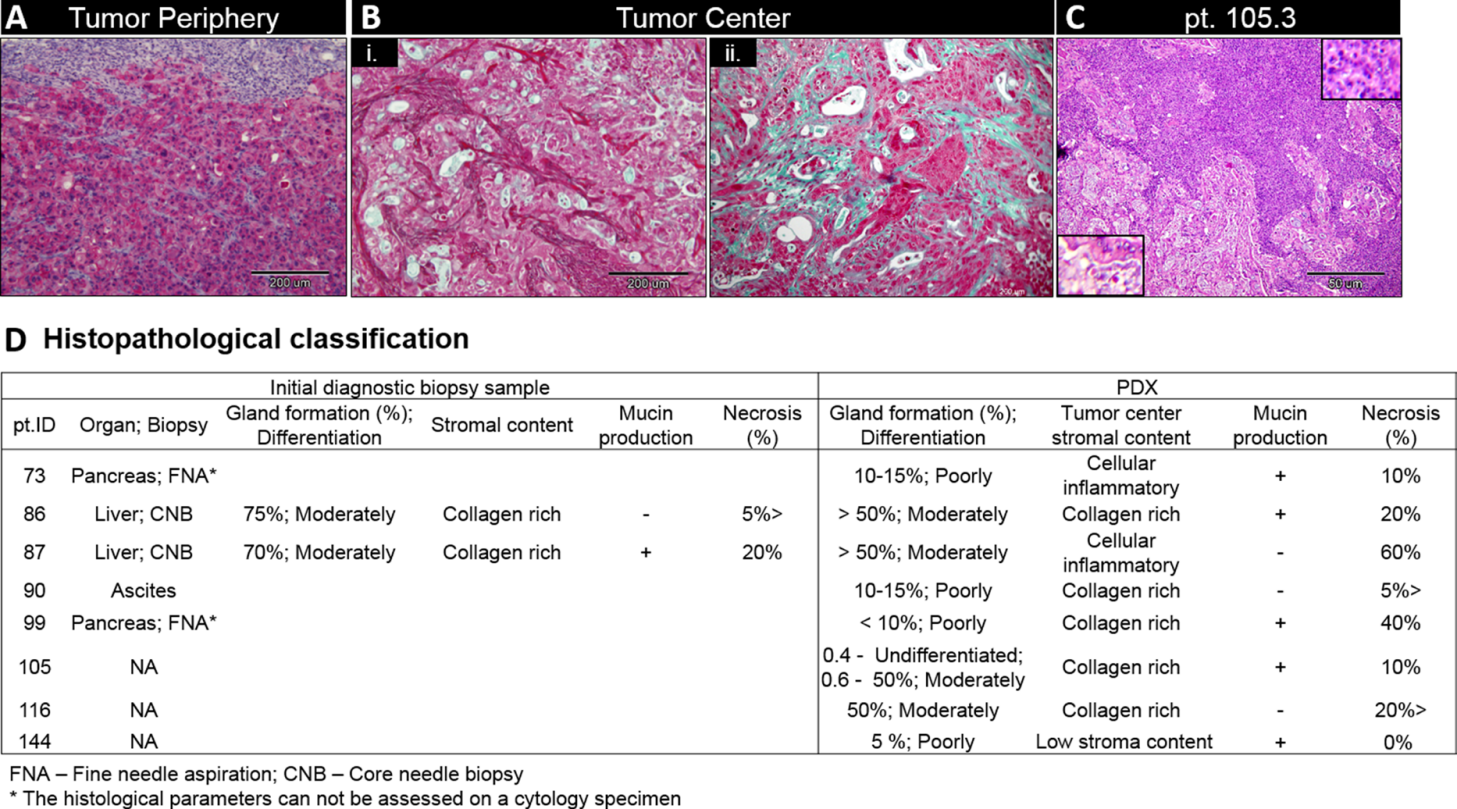
Stroma characterization and PDX classification (**A**) Stroma content in PDX periphery (pt. 99). (**B**) PDX center demonstrating cellular inflammatory stroma (i, pt. 87) and enriched collagen stroma (ii, pt. 86). (**C**) H&E staining of patien 105 F1 generation PDX demonstrating two distinct growth patterns - moderately differentiated adenocarcinoma (bottom inset) and undifferentiated growth (top inset). Scale bar 200 μm for A-C; 50 μm - D. (**D**) Diagnostic biopsies and PDX histopathological classification

Desmoplastic stroma was evident in all PDXs however the type and percentage of stroma varied between tumors. In all PDXs, the periphery of the tumors tended to have a cellular inflammatory interface with the mouse subcutaneous tissue (Figure 3A) while the center of the tumors had a paucity of inflammatory cells. Two distinct growth patterns of stroma were observed: 1. Inflammatory cellular stroma (Figure 3Bi) (*n* = 2) 2. Collagen rich cell poor stroma (Figure 3Bii) (*n* = 5).

PDX's tumors were graded as poorly (*n* = 4) and moderately (*n* = 3) differentiated; one PDX (patient 105) demonstrated two growth patterns: moderately differentiated adenocarcinoma and undifferentiated tumor growth (Figure 3C). In 5/6 PDXs, the histologic parameters were consistent throughout serial passaging. In the one PDX with the bi-phasic growth pattern seen on generation F1 (pt. 105), subsequent passages showed only the moderately differentiated portion of the tumor.

Of note, the PDAC ascites/pleural effusion PDXs accurately recapitulated the phenotypic features of PDAC primary tumors.

### Short tandem repeat (STR) profiling

DNA from F1 generation PDX and matching patients’ germline DNA (*n* = 8) were isolated and STR at 16 different loci were compared. All samples were classified in 80%-100% match range, reflecting that both samples are related (same donor). All PDXs had LOH in one to seven loci (median-3) (Supplementary Figure 1A). Chromosomal gain of one locus was observed in two PDX samples (pt. 105, pt. 144). Chromosomal loss or deletions within the Y chromosome were observed in three male patients’ (pt.86, pt.90 and pt.105) PDXs. Loss of the Y chromosome has also been observed in a number of patient tumors in various cancer types [27–29, 30]. Overall, genetic identity between our PDXs and matching germline is preserved.

### *In vitro* establishment of PDX-derived tumor cell cultures

**Figure 4:**
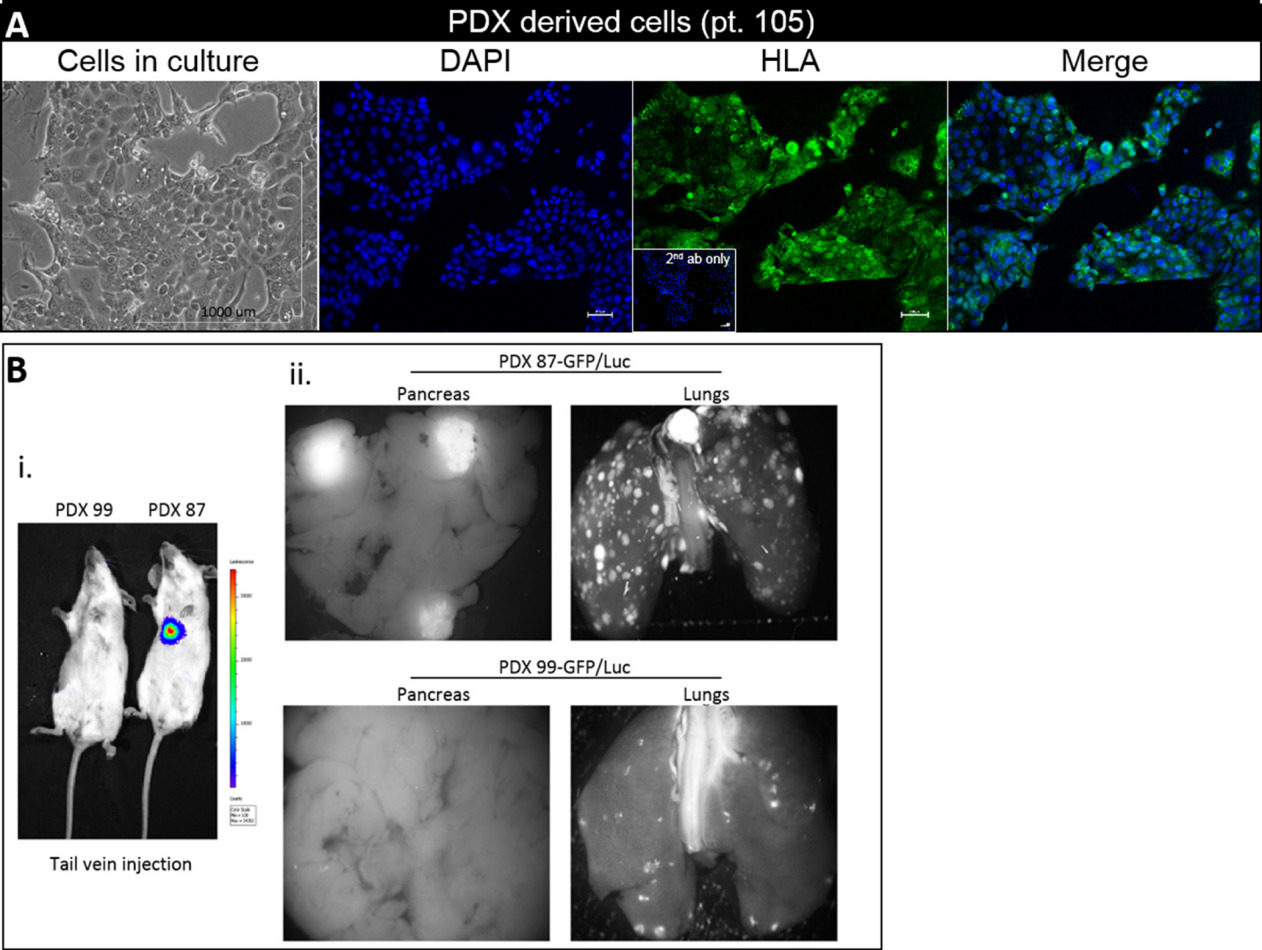
PDX derived cultures and metastatic PDX model (**A**) Photomicrograph of representative PDX derived cells. Cells grown in culture (left; scale bar 1000 μm), nuclear DAPI staining (second), positive HLA-A staining (third) (inset - merged of 2^nd^ ab only stained cells), and merged photos (right). For immunofluorescence images - magnification × 10, scale bar–100 μm (**B.i**) IVIS imaging demonstrating metastatic growth of PDX 87/GFP-LUC in lungs region of NOG mice (**B.ii**) Representative fluorescence images of lungs and pancreas from PDX 87/GFP-Luc (upper panel) and PDX 99/GFP-Luc group (lower panel). No liver and spleen metastases were detectable with either cells.

PDXs were excised, and digested to single cells. Initially, a mixed population of epithelial and stromal cells was observed. Epithelial cells were purified and PDX-derived cultures displayed a typical cuboidal epithelial-like morphology and formed monolayer cells congregated in islands. HLA positive staining of PDX derived culture indicated human origin (Figure 4A). Ascites cultures served as positive control (Supplementary Figure 1B).

Doubling time of PDX-derived cells ranged between 38 to 70 hours, compared to 32 to 49 hours of the established cell lines (Panc1, AsPC1 and Capan1) (data not shown). Cultures remain stable in doubling time and morphology for more than 20 passages. Additionally, cultures are being routinely authenticated and stained for EpCAM (data not shown).

### Establishment of a metastatic PDX model

PDX- derived cells from two different patients (pt. 87 and 99) were stably transfected with Green Fluorescent Protein (GFP) and Luciferase (see methods section). We determined the ability of the GFP-Luc transfected cells to form metastases in NOG (NOD/Shi-scid IL2rgamma(null) mice, utilizing a tail vein injection procedure. Eight weeks post injection, numerous metastases in the lung region were detected by IVIS (*In Vivo* Imaging System) in PDX 87-GFP/Luc animal group (*n* = 6), no sign of metastasis was identified in PDX 99-GFP/Luc group (Figure 4Bi). Mice were sacrificed and organs were harvested. Imaging by fluorescent binocular microscope for GFP showed numerous metastasis in lungs and pancreas of PDX 87-GFP/Luc group (Figure 4Bii, upper panel), while in PDX 99-GFP/Luc group we saw reduced number of detectable metastatic nodules in lungs and no metastases were observed microscopically in the pancreas (Figure 4Bii, lower panel).

### Whole genome sequencing

To investigate whether the emergent tumors in ascites/pleural effusion PDXs reflect the primary pancreatic cancer tumors and to gain insights into genomic molecular alterations of advanced PDAC disease, we performed whole genome sequencing on both the (F1) xenograft from each individual as well as DNA obtained from patient's blood (*n* = 6). The xenograft genome was compared to the matched normal blood genome in order to identify somatic mutations.

We detected a median of 6250 (range 2059–7688) somatic SNV alterations in our PDX models. 237 non-synonymous somatic SNVs were identified across all PDXs, including 11 stopgains, with a median of 38 non-synonymous somatic variants per PDX (range 12–65). A total of 5 splice altering SNVs were also detected (range 0–3).

The most common substitution was the C > T transition, which accounted for 38% of somatic SNVs detected. Each sample was dominated by C > T substitutions (ranging from 33%-43%) which is consistent with the Age Related mutational signature commonly observed in other PDAC samples [31].

The median number of small somatic insertions and deletions (INDELs) detected in all samples was 587 (range 234–905). Somatic insertions ranged from 146 to 580 with a median of 327 variants identified, and somatic deletions ranged from 88 to 325 with a median of 267 variants identified. 94% of insertions and 76% of deletions affected 3 or fewer base pairs (insertions ranged from 90% to 97%, deletions from 74% to 80%). 15 somatic INDELs resulting in frameshifts were detected in the samples (range 0–5) and one sample (pt. 99) had 2 non-frameshift variants.

Neo-antigen load (the number of novel peptides created by somatic SNVs and INDELs and predicted to have a high affinity to the HLA types identified in the patient) ranged from 18 in pt. 90 to 58 in pt. 105, with a median of 39.5, which is in line with the median neo-antigen load of 48 for a series of 160 PDAC whole genomes [6].

**Figure 5:**
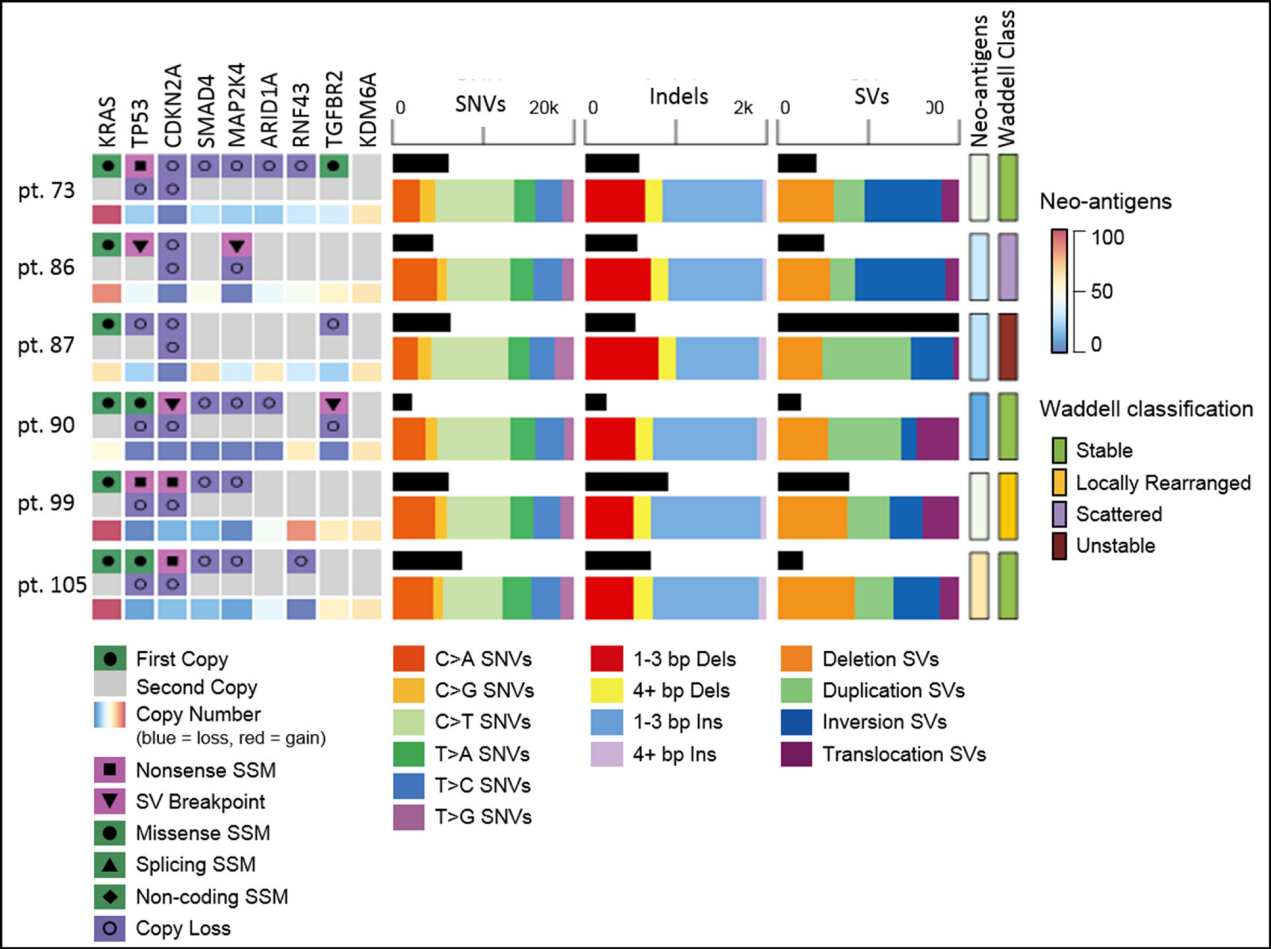
Whole genome sequencing of ascites/pleural effusion PDX Mutations in key PDAC genes in ascites/pleural-PDX tumors. Type of alteration as indicated. Copy number of each gene indicated in the third line per sample. Mutational load (black horizontal column) of SNVs, INDELs and SVs; alteration type as indicated. Neo-antigens load estimation and PDAC classification by Waddell *et al*. for each ascites/pleural-PDX. SSM - somatic single-base mutation; SV- structural variation; SNVs–single nucleotide variants; bp- base pair; Ins–insertion; Dels- deletions.

We identified 448 structural variations across all PDXs, with a median of 52 per PDX sample (range 25–223). Deletions larger than 1 kilobase, duplications, and inversions accounted for 131, 158, and 118 events, respectively. 41 Translocations were identified. We classified the samples based on the pattern of structural rearrangements according to the Waddell et al. system [5]; Three cases were Stable (pt. 73, pt. 90 and pt. 105), one was Locally Rearranged (pt. 99), one was Scattered (pt. 86) and one was Unstable (pt. 87). The Unstable class tumor was enriched with duplication variants, as it contained 70% of the SVs of that type observed in any of the 6 -PDX samples (Figure 5).

In total, 254 variants were found to change the protein-coding sequence in 239 genes, including 5 that are significantly mutated in PDAC (KRAS in six, TP53 in four, CDKN2A in two, TGFBR2 in one and U2AF1 in one PDX). In addition, of the 448 structural variants detected, 297 had breakpoints within the transcript of genes and four PDAC genes were affected (TP53, CDKN2A, TGFBR2 and MAP2K4). Commonly found (>2 samples) SNV and INDELs across all PDXs is presented (Supplementary Table 2).

The most common mutated gene was KRAS (G12D, *n* = 3; G12V, *n* = 2; Q61H, *n* = 1). These mutations were verified by Sanger sequencing (data not shown). KRAS was also found to be amplified in 3 of the 6 samples. The second most commonly mutated gene was TP53, which was somatically altered in 5/6 PDXs and also underwent loss of one copy in 5/6 cases. CDKN2A appeared to be biallelically inactivated in all six cases; three by mutation and loss of heterozygosity, and three by two copy deletion. SMAD4 was not found to be mutated in any of the PDXs, but did lose a single copy of the gene in four of six samples.

PDX WGS was compared to mutation panel from primary diagnostic biopsy (EUS FNA) of pt. 99. Three non-synonymous mutation were demonstrated in primary biopsy: KRAS, PIK3CA and FLCN; all mutations were identified in ascites PDX (Supplementary Table 3).

### PDX classified with an “unstable subtype”

Recent sequencing efforts have divided PDAC tumors into potential therapeutic subtypes [32, 6]. One subtype (unstable genome) is enriched with tumors demonstrating DNA damage repair deficiency (DDR subtype) [5]. The therapeutic relevance of these subtypes in advanced disease has not been thoroughly investigated.

**Figure 6:**
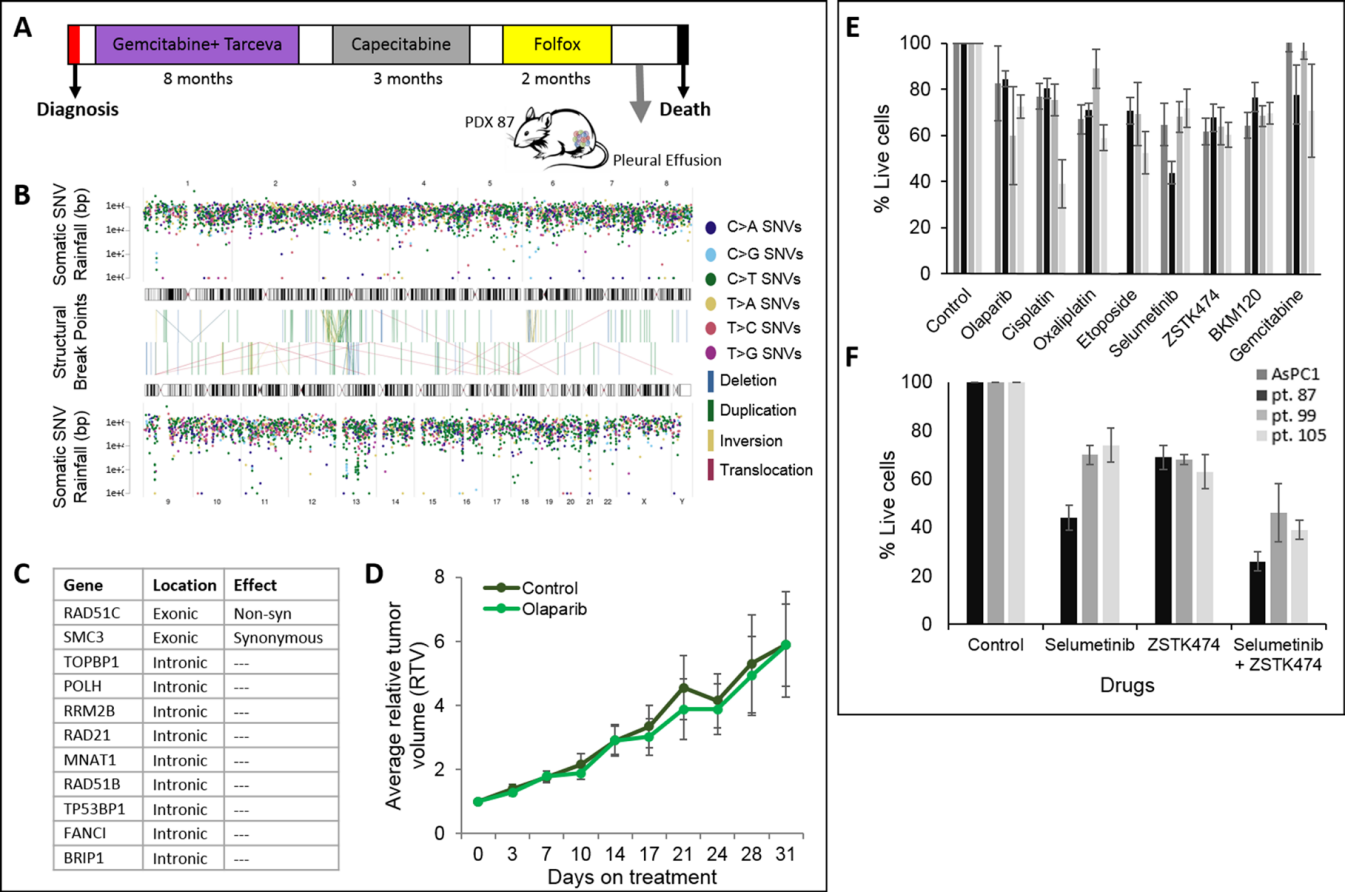
PDX with an unstable genome and *in vitro* and *in vivo* sensitivity assay (**A**) Clinical course illustration of patient 87. (**B**) Somatic SNV ‘Rainfall’ and SV plot for PDX 87. Each dot represents a single somatic mutation according to position in the genome (horizontal axis). Vertical axis denotes the genomic distance of each mutation from the previous mutation. Structural variants are shown in the center of the image as lines connecting their breakpoints. (**C**) Homologous recombination deficiency (HRD) altered genes in patient 87 PDX. (**D**) *In vivo* response to treatment of PDX 87. Average relative tumor volume as a function of days post treatment initiation (olaparib; 50 mg/kg; IP) (light green) and control (dark green) (*n* = 6/group). (**E**, **F**) *In vitro* chemo sensitivity of AsPC1 and PDX derived cells (pt. 87, pt.99 and pt.105) to targeted drugs: olaparib (8 ug), cisplatin (2 uM), oxaliplatin (1.2 uM), etoposide (8 nM), selumetinib (0.5 uM), ZSTK474 (0.5 uM), BKM120 (0.5 uM) and gemcitabine (4 nM). F. Combination treatment of selumetinib (0.5 uM) and ZSTK474 (0.5 uM); Mean ±SD of 3 independent experiments.

One of our PDX models demonstrated an unstable genome, generated from patient with a favorable clinical response. Patient # 87 was a 66-year-old male, diagnosed with stage IV PDAC with liver metastases. He received three therapeutic lines (Supplementary Table 1) and demonstrated a superior OS of 17 months (Figure 6A). PDX 87 WGS data displayed mutational load of 6,451 SNV and considerably high load of structural variations, enriched with duplications (Figure 6B) and classified as an unstable genome subtype of Waddell et al. [5]. A non-synonymous somatic RAD51C variant was identified (c.C236G) in WGS. RAD51C is involved in DNA repair and germline RAD51C mutations are associated with Fanconi-anemia like syndrome. We are uncertain whether this particular variant generates a functionally defective protein in PDAC though we note the Waddell unstable genome in this case. Additional alterations in Homologous recombination deficiency (HRD) genes were observed in pt. 87 PDX (Figure 6C).

### *In vitro* and *in vivo* sensitivity assays

PDX-derived cells generated from three different patients were screened with a spectrum of chemotherapies and biological available compounds. A reduction of ≥ 40% of tumor cells was only demonstrated in selected PDX derived cells as following: olaparib (pt. 99), cisplatin (pt. 105) and selumetinib (pt. 87) (Figure 6E). Additionally, a synergistic effect was observed with selumetinib and ZSTK474 combination (pt. 87) (Figure 6F). Pleural effusion from patient 87 (unstable genome) was obtained following progression on third line treatment and a PDX model was established from advanced clinical disease, resistant to platinum (Figure 6A). No tumor attenuation was demonstrated *in vivo* with PARPi (olaparib; 50 mg/kg/day) treatment (*n* = 6) as compared to control (*n* = 6) (Figure 6D). Additionally, chemo-resistance was observed *in vitro* to PARPi, cisplatin and oxaliplatin (Figure 6E).

## DISCUSSION

We previously described the characterization of ascites derived PDAC cells (ref; Golan). Herein, we present a rapid and novel xenograft model utilizing highly chemo-resistant metastatic PDAC cells. Harnessing these cancer cells from a palliative procedure for generation of PDX provides an opportunity for the study of resistant metastatic PDAC *in vivo*.

The ascites/pleural effusion PDXs, generated from single cells in suspension, developed glandular forming mucin producing PDAC, demonstrating the multi-pluripotent nature of these cells. The PDX tissues retain morphology, architecture, and molecular signatures similar to original PDAC tumors. Variation in tumor growth kinetic was observed between patients, the latter may rise from initial differences in the number of malignant cells in the milieu and from clonal heterogeneity. No spontaneous metastases nor ascites/pleural effusion dispersing was observed in the subcutaneous model. Preliminary experiments utilizing GFP labeled PDX derived cells for generation of a metastatic model has shown spontaneous lung metastases (Figure 4B).

Desmoplasia is a prominent characteristic of PDAC which contains extensive stroma that constitutes up to ~80–90% of the tumor volume [33]. An extensive desmoplastic reaction was observed in all PDXs. Some cases show a more extensive stroma-rich and lymphocyte-poor reaction and others the opposite. The single cancer cells derived from ascites fluid and pleural effusion were able to form tumors with a significant desmoplastic reaction in a similar fashion to solid pancreatic masses. The clinical immunotherapeutic relevance of these findings should be further evaluated in alternative models.

Extensive efforts regarding WGS analysis of PDAC has been undertaken, however the majority of the information gathered has been from primary tumors [5, 32, 6]. Our study attempts to rigorously identify, expand and perform WGS on advanced therapeutic resistant tumor cells to develop a platform to test drug combinations and specifically discover resistance pathways. The PDXs genomic analysis display significant overlap with available sequencing data on primary tumors [34]. The driver mutations are identified in early stage of disease and remain constant during disease progression. For example: PDX 87 displays an unstable genome. Of importance, the classical BRCAness phenotype [7] accumulates small deletions (3 bp–10 kbp) and also shows a point mutation signature [5], these features were not seen in this case despite the ‘unstable genome’. This patient's clinical scenario and his genomic analysis demonstrating accumulation of duplications may be a result of some yet to be established DNA repair deficiency. The unstable genome may be in the spectrum of DDR as evidenced by an initial good response to chemotherapy, yet it does not fully satisfy mutational signature. The therapeutic relevance of his unstable phenotype may have changed over the course of his disease. *In vivo* and *in vitro* results from clinical resistant disease demonstrate corresponding resistance to platinum/PARPi agents. This suggests that alternative biomarkers for identification of the relevant resistant clinical scenario beyond WGS need to be developed. Transcriptome and proteome data in advanced stages of disease may be more informative for clinical decisions.

The advantage of utilizing ascites fluid and pleural effusion cells relies on the noninvasive procedure and the viability of these sources as it is routinely being performed for patient's clinical comfort. Additionally, these cells recapitulate the most aggressive state of disease. Liver core needle biopsies are usually being performed prior to treatment initiation and therefore might not always reflect the chemo resistant state of disease. Ideally, these models and PDX from primary tumors should be generated and tested in parallel.

The limitations of our model include the inability to study tumor cell-host immune response interactions in the immune-deficient mice background. In addition, the cells identified and grown in the PDX model are captured from the peritoneal cavity and pleural effusion and it is necessary to further estimate to what extent they can represent additional metastatic sites such as lung or liver metastasis.

In summary, our results show remarkable similarity of ascites/pleural effusion PDX to early stage PDAC. The strength of our sample collection includes non-invasive procedure, collecting tumor cells at advanced disease state, allow the modelling of the refractory clinical scenario, which is lacking in comparative models to date. These models need to be further investigated at the transcriptome and protein expression level and interrogated to identify mechanisms of resistance that rapidly occurs in PDAC in the clinical setting.

## MATERIALS AND METHODS

### Ethic statement

Ascites and pleural effusion fluids were obtained following approval of Sheba Medical Center institutional ethics committee with consent from patients. Conduct of the research project was approved by the Israeli Ministry of Health and the study was performed with strict accordance to animal use protocols approved by the institutional animal care committee.

### Ascites fluid and pleural effusion collection and patient's description

Two to five liters of fluids were collected at the time of paracentesis/thoracentesis for palliative reasons from PDAC patients. The acquired fluid was used in parallel for cytology evaluation and *in vivo* xenograft generation. Fifty ml fluid was sent for cytology analysis (see below). The remaining fluids were used directly for generation of PDAC PDX.

Data on patient demographics, clinical history, surgery, systemic treatment, response to treatment and BRCA 1/2 germ-line mutation status were collected from the Sheba Medical Center cancer database and patient records. Clinical stages were classified according to the TNM AJCC staging system. Response to treatment was categorized according to imaging results.

### Cell lines

Human pancreatic carcinoma cell lines Panc1, Capan1 and AsPC1 were used and authenticated by STR profiling according to international cell line authentication committee (ICLAC) (ANSI/ATCC ASN-0002-2011) and mycoplasma free (EZ-polymerase chain reaction (PCR) Mycoplasma test kit; Biological Industries). Cell were cultured in full RPMI-1640 media. 3 × 10^6^ cells were subcutaneously transplanted into flanks of Hsd:athymic nude-Foxn1nu immunodeficient mice (Harlan) for xenograft establishment.

### PDAC patient derived xenograft model

Ascites fluid or pleural effusion were spun at 1200rpm for 10 minutes. Pellets were subcutaneously injected into the flanks of 6–8 week old nude mice for establishment of first generation (F1) xenograft (*n* = 1–2/model). Tumors reaching maximum volume (1.5 cm^3^) were harvested, cut into ~1–2 mm^3^ pieces and serially transplanted to new recipient mice (*n* = 3–4). All PDXs were successfully passaged at least twice to determine the ability to perpetuate the models. Transplantations were performed under anesthesia by intraperitoneal injection of Ketamine (100 mg/kg) and Xylazine (10 mg/kg). Following resection of the tumors, a 4 mm incision was used to access the subcutaneous area of the sterilized anesthetized recipient mice and a ~1–2 mm^3^ tumor piece was inserted. Incisions were sutured by surgical clips (9 mm auto clips, MikRon Precision Inc.) and animals were monitored for signs of distress, wound dehiscence or signs of infection. Tumor volume was followed bi-weekly by digital caliper according to the formula (length × width^2^)/2 and tumor growth kinetics curves were generated. Mice were weighted twice weekly.

Mice were housed in specific pathogen-free conditions, acclimatized to their new surroundings for at least 48 hours prior to tumor engraftment, and maintained in accordance with institutional standards. Mice bearing 1.5 cm^3^ tumors were humanely sacrificed using CO_2_ inhalation and tumor samples, liver, pancreas and spleen were excised. Tumor samples were fixed in 4% formaldehyde, digested into single cells for generation of PDX-derived cell lines, cut into 2mm^3^ pieces and snap frozen in liquid nitrogen and cryopreserved in fetal bovine serum (FBS) with 10% DMSO.

### Histopathological characterization of PDAC fluids and PDX tumors

Fluid cytological cell blocks were prepared by fixation in 1:1 4% paraformaldehyde and 96% ethanol. Slides were stained with hematoxylin-eosin (H&E) and immunostained for epithelial markers: BerEp4 (1:50; DASO M 0804), Muc1 (1:50; CM 290M-16), CEA (1:100; Zymed 18-0013), TAG-72 (1:100; Cell marque CMC-897) and the mesothelial marker calretinin (1:20; CM 232A-76). Slides were reviewed and classified as malignant/non-malignant according to cell morphology and immunostaining.Formalin-fixed paraffin-embedded tumor sections were stained with H&E and Masson's trichome and immunostained with human leucocyte antigen (HLA-A) (1:200; EP1395Y; abcam 52922). All slides were reviewed by a pathologist with expertise in PDAC and assessed for tumor architecture and desmoplastic tumor response. Slides were classified accordingly: 1. Differentiation (poorly; moderately and well differentiated adenocarcinoma) 2. Stromal cellularity: i). Inflammatory cellular stroma ii). Enriched collagen cell poor stroma 3. Mucin production (yes/no) and 4. Necrosis (%).

### Short tandem repeat (STR) profiling

Genomic DNA was extracted using the Qiagen DNA Easy Blood and Tissue Kit (Cat #69506) and was genotyped for 15 Autosomal STR loci and Amelogenin (X/Y) using Promega Power Plex16HS PCR kit (Cat#DC2101). STR analyzes was assigned using Soft Genetics, Gene Marker Software Version 1.85. Alleles were matched to STR profile of matching germline DNA for PDX and ATCC for cell lines. A minimum of 80% match threshold was used to indicate a shared genetic history (ANSI/ATCC ASN-0002-2011. Authentication of Human Cell Lines: Standardization of STR Profiling. ANSI eStandards Store, 2012. Cell line authentication was provided by Elizabeth Cox and the team at University of Arizona Genetics Core (via Science Exchange).

### Ascites/pleural effusion PDX digestion and *in-vitro* experimental design

Tumor digestion and generation of PDX-derived cell lines: F3-F4 generation PDXs were minced into single-cells by enzymatic digestion with 0.2% collagenase type-I (Sigma Aldrich) for 2 h in 37°C. The cells were then strained through a 70 uM nylon mesh, washed and cultured in full RPMI medium. Short trypsinization was performed in order to isolate tumor cells from mouse stromal cells. The less adherent mouse stromal cells were washed and remaining epithelial cells were re-cultured in complete media. The procedure was performed several times until a pure epithelial population was obtained.

### Room temperature

### Sulforhodamine B (SRB) cytotoxicity assays

SRB assays were performed as previously described [35]. In brief, PDX-derived cells and established cell lines were seeded at a density of 5 × 10^3^ cells/well in 96 well plates in complete medium. The following day cells were treated with therapeutic agents and combinations: olaparib (AZD2281) 8 ug, cisplatin 2 uM, oxaliplatin 1.2 uM, taxol 1 nM, gemcitabine 4 nM, etoposide 8 nM, selumetinib (AZD6244) 0.5 uM, buparlisib (BKM120) 0.5 uM and ZSTK474 0.5 uM. After 72 h, cells were fixed with 10% trichloroacetic acid and stained with 0.057% SRB. The absorbance was measured using microplate reader at 510 nm. Each concentration was tested in triplicate, and results shown are representative of 3–4 independent experiments.

### Doubling time

Each PDX-derived cell line was seeded (5×10^3^ cells per well) in six 96-well plates. The first plate was used as the time zero plate. The remaining plates were incubated at 37°C and removed from the incubator every 24 h (24–120 h) and SRB assay was performed. Growth curves were plotted, and the doubling time was calculated. (Roth V. 2006 Doubling Time Computing, Available from: http://www.doubling-time.com/compute.php).

### Therapeutic evaluation *in vivo*

All animal studies were approved by the Sheba Medical Center Institutional Animal Care and Use Committee (Helsinki 930/14). Preserved frozen tumors from PDX 87 were thawed and implanted to a group of animals. When tumors reached palpable size (~100 mm^3^), mice were randomized into the following treatment groups (*n* = 6/group): (a) PARPi (Olaparib, Selleckchem) dissolved in 10% Hydroxypropyl-β-Cyclodextrin (Sigma), 50 mg/kg, intra-peritoneal (I.P.) (b) Vehicle control (10% Hydroxypropyl-β-Cyclodextrinl; IP); five days a week for four weeks.

Tumor volumes were measured twice a week using a caliper according to the formula (length × width^2^/2). Mice were weighted twice a week. Mice were euthanized if the tumor burden reached 1.5 cm^3^ or the animals presented over 20% of the initial body weight loss. At the end of the experiments, animals were sacrificed by CO2 inhalation. Tumor growth curves are presented as average relative tumor volume (RTV) ± SE at each measurement point, for each treatment group.

### Metastatic PDX model

### Lentiviral vector production

High titer lentiviral vector stock was produced in human embryonic kidney (HEK) 293T cells following transient transfection with the vector pCCL.sin.cPPT.polyA.CTE.eGFP.minhCMV.hPGK.Luciferase.Wprep (10 μg). Lipofectamine 2000 was used for transfection and the plasmid was a kind gift from Prof.Carola Ponzetto (CeRMS University, Turin, Italy). The following packaging vectors were used: pMDLg/pRRE (5 μg), pRSV-REV (2.5 μg) and pMD2.VSVG (5 μg). Supernatants were harvested after 32 h, filtered through 0.22 μm pore size filters (Millipore, Billerica, MA) and used directly for subsequent target cell infection. PDX 87 and PDX 99 derived cells (1 × 105 ) were plated 16 hours beforehand (in 35-mm diameter culture dishes) and were transduced by the above described viruses to obtain PDX 87/GFP-Luc and PDX 99/GFP-Luc cells. The medium was changed 16 hours post infection and cells were kept in culture for 48 h before flow cytometry sorting of GFP positive cells. Luciferase activity from the same PDX cells was measured by bioluminescence imaging and confirmed with cell lysates luciferase assay. In sequential noninvasive imaging, attached PDX cells were exposed to 15 mg/mL of luciferin directly supplemented in the serum free medium and detected with a cooled charge-coupled device (CCD) bioluminescence camera (*In Vivo* Imaging System, IVIS 50; Xenogen, Alameda, CA) immediately. Photon emission was acquired for 120 seconds and bioluminescence was quantified using Living Image software (Xenogen Corp., Alameda, CA). To confirm the results, *ex vivo* luciferase activity of both PDX clones was tested by the Dual Luciferase Assay kit purchased from Promega (Madison, WI). Cells (4 × 10^5^) were extracted with lysis solution and 50 μl of cell suspension was added to 100 μl of luciferin mix. The luciferase activity was measured by a VictorX luminometer.Lentiviral vector production

### Animal model

All animal studies were approved by the Weizmann Institute's Animal Care and Use Committee. PDX 87/GFP-Luc and PDX 99/GFP-Luc derived cells (3.5 × 10^6^ /100 ul) were injected to tail vein of female 4 week old NOG mice (Jackson Laboratory, USA). Mice were monitored twice weekly for the evidence of morbidity related to pulmonary metastases.

### Metastasis test in animals

IVIS (Xenogen Corp., Alameda, CA) consists of a highly sensitive, charge-coupled digital camera that captures photons of light emitted by cells that have been engineered to produce bioluminescence in the living animal. The substrate luciferin was injected into the intraperitoneal cavity at a dose of 150 mg/kg body weight (15 mg/ml luciferin), approximately 5 minutes before imaging. Mice were anesthetized with isoflurane/oxygen and placed on the imaging stage. Ventral images were collected for 30–60 seconds using the IVIS and quantified using Living Image software (Xenogen Corp., Alameda, CA). To check micrometastasis in lung, liver, spleen and pancreas were removed and washed, and images were acquired using a fluorescent binocular microscope (Olympus, USA).

### Whole genome sequencing of PDXs

50ng of Qubit (Life Technologies, Carlsbad, CA, Cat #Q32854) quantified gDNA was sheared to 550 bp fragments using the Covaris S2 Ultra-sonicator (Covaris Inc., Woburn, MA, USA) followed by 1x volume AMPure XP SPRI bead clean-up (Beckman Coulter Genomics, Danvers, MA, USA Cat#A63881). Libraries were constructed using enzymatic reagents from KAPA Hyper Library Preparation Kits (KAPA Biosystems, Woburn, MA USA Cat# KK8505) according to protocols described by Fisher, et al. [36] for end repair, A-tailing, and adapter ligation using NextFlex DNA barcodes (Bioo Scientific, Austin TX, 78744, Cat #514104). Adapter- ligated libraries were enriched using optimized PCR conditions by adding 6 μL of Illumina paired-end enrichment primers (Integrated DNA Technologies, Coralville, Iowa, USA), 75 μL of 2× KAPA HiFi HotStart ReadyMix (KAPA Biosystems, Woburn, MA, USA Cat#KK2602) and 33 μL of nuclease-free water (Life Technologies, Carlsbad, CA, USA Cat#AM993) to 36 μL of eluted DNA and amplified across 3 individual PCR reaction tubes. Libraries were incubated in Verti 96-well Thermal Cyclers (Life Technologies, Carlsbad, CA, USA) for 45s at 98°C and cycled 9 times for 15s at 98°C, 30s at 65°C, and 30s at 72°C. Following a 0.6x SPRI bead clean-up, post-PCR enriched libraries were eluted in 25 uL of elution buffer (Qiagen, Hilden Germany, Cat#19086) and validated using Agilent TapeStation High Sensitivity DNA Kit (Agilent Technologies, Santa Clara, CA, USA Cat#5067-5584, 5067-5585).

Libraries were quantified on the Illumina Eco Real-Time PCR Instrument (Illumina Inc., San Diego, CA, USA) using KAPA Illumina Library Quantification Kits (KAPA Biosciences, Woburn, MA, USA Cat#KK4835) according to the manufacturer's standard protocol. Paired-end cluster generation and sequencing was carried out for all libraries on the Illumina HiSeq 2500 platform using high-throughput 2×126 cycles (Illumina Inc., San Diego, CA, USA Cat #PE-401-4001/FC- 401-4001). Samples were sequenced to a minimum collapsed coverage of 35x× and 30× for xenograft and matched normal samples, respectively.

Once sequencing was complete, Xenome v1.01-r was used to identify and remove mouse reads. Human reads and ambiguous reads were aligned to hg19_random using BWA v0.6.2. Lanes were merged and PCR duplicates were flagged using Picard v1.90.

Somatic SNVs and INDELs were identified by comparing the xenograft and matched normal genomes using Strelka v1.07 and MuTect v1.1.4 and annotated with dbSNP 142, COSMIC v54, ANNOVAR v2013-06-21 and FunSeq v0.1. In order to increase precision, only SNVs that were called by both Strelka and MuTect were considered [37]. A blacklist consisting of SNVs and INDELs generated by aligning model mouse DNA to hg19_random was used to remove potential false positives caused by unfiltered mouse DNA.

Neo-antigen load was estimated by predicting the binding affinity between the HLA types derived from the matched normal sequencing with the peptides that result from the non-silent somatic variants observed in the tumor. POLYSOLVER v1.0 was used to determine HLA types. A perl-script was used to convert non-silent SNVs and INDELs to peptides with 9 amino acids. The binding affinity between the HLA types and peptides was predicted using NetMHC pan-2.8; any peptide with a binding affinity of < 500 Nm was considered to be a neo-antigen.

GATK v1.3.16 was used to realign, recalibrate and genotype the matched normal DNA by following the best practice guidelines made available by the Broad. Germline SNVs and INDELs were annotated with dbSNP 142, COSMIC v54, ANNOVAR v2013-06-21 and FunSeq v0.1.

Somatic structural variants (SVs) were called by applying CREST vAlpha and DELLY v0.5.5 to the xenograft and matched normal genomes. The output of each tool was filtered to remove false positives and the resulting lists of variants were merged. Somatic copy number variants (CNVs) were called by HMMCopy v0.1.1.

## SUPPLEMENTARY MATERIALS FIGURES AND TABLES



## References

[1] Conroy T, Desseigne F, Ychou M, Bouche O, Guimbaud R, Becouarn Y, Adenis A, Raoul JL, Gourgou-Bourgade S, de la Fouchardiere C, Bennouna J, Bachet JB, Khemissa-Akouz F (2011). FOLFIRINOX versus gemcitabine for metastatic pancreatic cancer. N Engl J Med.

[2] Goldstein D, El-Maraghi RH, Hammel P, Heinemann V, Kunzmann V, Sastre J, Scheithauer W, Siena S, Tabernero J, Teixeira L, Tortora G, Van Laethem JL, Young R (2015). nab-Paclitaxel plus gemcitabine for metastatic pancreatic cancer: long-term survival from a phase III trial. J Natl Cancer Inst.

[3] Peery AF, Crockett SD, Barritt AS, Dellon ES, Eluri S, Gangarosa LM, Jensen ET, Lund JL, Pasricha S, Runge T, Schmidt M, Shaheen NJ, Sandler RS (2015). Burden of Gastrointestinal, Liver, and Pancreatic Diseases in the United States. Gastroenterology.

[4] Grapsa D, Saif MW, Syrigos K (2015). Targeted therapies for pancreatic adenocarcinoma: Where do we stand, how far can we go?. World J Gastrointest Oncol.

[5] Waddell N, Pajic M, Patch AM, Chang DK, Kassahn KS, Bailey P, Johns AL, Miller D, Nones K, Quek K, Quinn MC, Robertson AJ, Fadlullah MZ (2015). Whole genomes redefine the mutational landscape of pancreatic cancer. Nature.

[6] Connor AA, Denroche RE, Jang GH, Timms L, Kalimuthu SN, Selander I, McPherson T, Wilson GW, Chan-Seng-Yue MA, Borozan I, Ferretti V, Grant RC, Lungu IM (2016). Distinct Mutational Signatures Are Associated With Correlates Of Increased Immune Activity In Pancreatic Ductal Adenocarcinoma. Jama Oncology.

[7] Alexandrov LB, Nik-Zainal S, Wedge DC, Aparicio SA, Behjati S, Biankin AV, Bignell GR, Bolli N, Borg A, Borresen-Dale AL, Boyault S, Burkhardt B, Butler AP (2013). Signatures of mutational processes in human cancer. Nature.

[8] Hughes P, Marshall D, Reid Y, Parkes H, Gelber C (2007). The costs of using unauthenticated, over-passaged cell lines: how much more data do we need?. Biotechniques.

[9] Hingorani SR, Wang L, Multani AS, Combs C, Deramaudt TB, Hruban RH, Rustgi AK, Chang S, Tuveson DA (2005). Trp53R172H and KrasG12D cooperate to promote chromosomal instability and widely metastatic pancreatic ductal adenocarcinoma in mice. Cancer Cell.

[10] Kojima K, Vickers SM, Adsay NV, Jhala NC, Kim HG, Schoeb TR, Grizzle WE, Klug CA (2007). Inactivation of Smad4 accelerates Kras(G12D)-mediated pancreatic neoplasia. Cancer Res.

[11] Aguirre AJ, Bardeesy N, Sinha M, Lopez L, Tuveson DA, Horner J, Redston MS, DePinho RA (2003). Activated Kras and Ink4a/Arf deficiency cooperate to produce metastatic pancreatic ductal adenocarcinoma. Genes Dev.

[12] Walters DM, Stokes JB, Adair SJ, Stelow EB, Borgman CA, Lowrey BT, Xin W, Blais EM, Lee JK, Papin JA, Parsons JT, Bauer TW (2013). Clinical, molecular and genetic validation of a murine orthotopic xenograft model of pancreatic adenocarcinoma using fresh human specimens. PLoS One.

[13] Garrido-Laguna I, Uson M, Rajeshkumar NV, Tan AC, de Oliveira E, Karikari C, Villaroel MC, Salomon A, Taylor G, Sharma R, Hruban RH, Maitra A, Laheru D (2011). Tumor engraftment in nude mice and enrichment in stroma- related gene pathways predict poor survival and resistance to gemcitabine in patients with pancreatic cancer. Clin Cancer Res.

[14] Kim MP, Evans DB, Wang H, Abbruzzese JL, Fleming JB, Gallick GE (2009). Generation of orthotopic and heterotopic human pancreatic cancer xenografts in immunodeficient mice. Nat Protoc.

[15] Tentler JJ, Tan AC, Weekes CD, Jimeno A, Leong S, Pitts TM, Arcaroli JJ, Messersmith WA, Eckhardt SG (2012). Patient-derived tumour xenografts as models for oncology drug development. Nat Rev Clin Oncol.

[16] Allaway RJ, Fischer DA, de Abreu FB, Gardner TB, Gordon SR, Barth RJ, Colacchio TA, Wood M, Kacsoh BZ, Bouley SJ, Cui J, Hamilton J, Choi JA (2016). Genomic characterization of patient-derived xenograft models established from fine needle aspirate biopsies of a primary pancreatic ductal adenocarcinoma and from patient-matched metastatic sites. Oncotarget.

[17] Gore J, Craven KE, Wilson JL, Cote GA, Cheng M, Nguyen HV, Cramer HM, Sherman S, Korc M (2015). TCGA data and patient-derived orthotopic xenografts highlight pancreatic cancer-associated angiogenesis. Oncotarget.

[18] Cree IA, Glaysher S, Harvey AL (2010). Efficacy of anti-cancer agents in cell lines versus human primary tumour tissue. Curr Opin Pharmacol.

[19] Fichtner I, Becker M, Zeisig R, Sommer A (2004). In vivo models for endocrine-dependent breast carcinomas: special considerations of clinical relevance. Eur J Cancer.

[20] Fiebig HH, Maier A, Burger AM (2004). Clonogenic assay with established human tumour xenografts: correlation of *in vitro* to *in vivo* activity as a basis for anticancer drug discovery. Eur J Cancer.

[21] Hidalgo M, Bruckheimer E, Rajeshkumar NV, Garrido-Laguna I, De Oliveira E, Rubio-Viqueira B, Strawn S, Wick MJ, Martell J, Sidransky D (2011). A pilot clinical study of treatment guided by personalized tumorgrafts in patients with advanced cancer. Mol Cancer Ther.

[22] Krumbach R, Schuler J, Hofmann M, Giesemann T, Fiebig HH, Beckers T (2011). Primary resistance to cetuximab in a panel of patient-derived tumour xenograft models: activation of MET as one mechanism for drug resistance. Eur J Cancer.

[23] Adam RA, Adam YG (2004). Malignant ascites: past, present, and future. J Am Coll Surg.

[24] Hicks AM, Chou J, Capanu M, Lowery MA, Yu KH, O'Reilly EM (2016). Pancreas Adenocarcinoma: Ascites, Clinical Manifestations, and Management Implications. Clin Colorectal Cancer.

[25] Puiffe ML, Le Page C, Filali-Mouhim A, Zietarska M, Ouellet V, Tonin PN, Chevrette M, Provencher DM, Mes-Masson AM (2007). Characterization of ovarian cancer ascites on cell invasion, proliferation, spheroid formation, and gene expression in an *in vitro* model of epithelial ovarian cancer. Neoplasia.

[26] Golan T, Atias D, Barshack I, Avivi C, Goldstein RS, Berger R (2014). Ascites-derived pancreatic ductal adenocarcinoma primary cell cultures as a platform for personalised medicine. Br J Cancer.

[27] Bianchi NO (2009). Y chromosome structural and functional changes in human malignant diseases. Mutat Res.

[28] Minner S, Kilgue A, Stahl P, Weikert S, Rink M, Dahlem R, Fisch M, Hoppner W, Wagner W, Bokemeyer C, Terracciano L, Simon R, Sauter G (2010). Y chromosome loss is a frequent early event in urothelial bladder cancer. Pathology.

[29] Wallrapp C, Hahnel S, Boeck W, Soder A, Mincheva A, Lichter P, Leder G, Gansauge F, Sorio C, Scarpa A, Gress TM (2001). Loss of the Y chromosome is a frequent chromosomal imbalance in pancreatic cancer and allows differentiation to chronic pancreatitis. Int J Cancer.

[30] Mattie M, Christensen A, Chang MS, Yeh W, Said S, Shostak Y, Capo L, Verlinsky A, An Z, Joseph I, Zhang Y, Kumar-Ganesan S, Morrison K (2013). Molecular characterization of patient-derived human pancreatic tumor xenograft models for preclinical and translational development of cancer therapeutics. Neoplasia.

[31] Connor AA, Gallinger S (2015). Hereditary Pancreatic Cancer Syndromes. Surg Oncol Clin N Am.

[32] Bailey P, Chang DK, Nones K, Johns AL, Patch AM, Gingras MC, Miller DK, Christ AN, Bruxner TJ, Quinn MC, Nourse C, Murtaugh LC, Harliwong I (2016). Genomic analyses identify molecular subtypes of pancreatic cancer. Nature.

[33] Hartel M, Di Mola FF, Gardini A, Zimmermann A, Di Sebastiano P, Guweidhi A, Innocenti P, Giese T, Giese N, Buchler MW, Friess H (2004). Desmoplastic reaction influences pancreatic cancer growth behavior. World J Surg.

[34] Yachida S, Jones S, Bozic I, Antal T, Leary R, Fu B, Kamiyama M, Hruban RH, Eshleman JR, Nowak MA, Velculescu VE, Kinzler KW, Vogelstein B (2010). Distant metastasis occurs late during the genetic evolution of pancreatic cancer. Nature.

[35] Vichai V, Kirtikara K (2006). Sulforhodamine B colorimetric assay for cytotoxicity screening. Nature protocols.

[36] Fisher S, Barry A, Abreu J, Minie B, Nolan J, Delorey TM, Young G, Fennell TJ, Allen A, Ambrogio L, Berlin AM, Blumenstiel B, Cibulskis K (2011). A scalable, fully automated process for construction of sequence-ready human exome targeted capture libraries. Genome Biol.

[37] Denroche RE, Mullen L, Timms L, Beck T, Yung CK, Stein L, McPherson JD, Brown AM (2015). A cancer cell-line titration series for evaluating somatic classification. BMC Res Notes.

