# Supplementary material for: Pancreatic cancer ascites xenograft–an expeditious model mirroring advanced therapeutic resistant disease
Source: Oncotarget. 2017 Apr 19;8(25):40778–90. doi: 10.18632/oncotarget.17253 (PMC5522335; doi:10.18632/oncotarget.17253)
Supplement: Supplementary file 1 [file oncotarget-08-40778-s001.pdf]

Pancreatic cancer ascites xenograft–an expeditious model mirroring advanced therapeutic resistant disease

Supplementary Materials

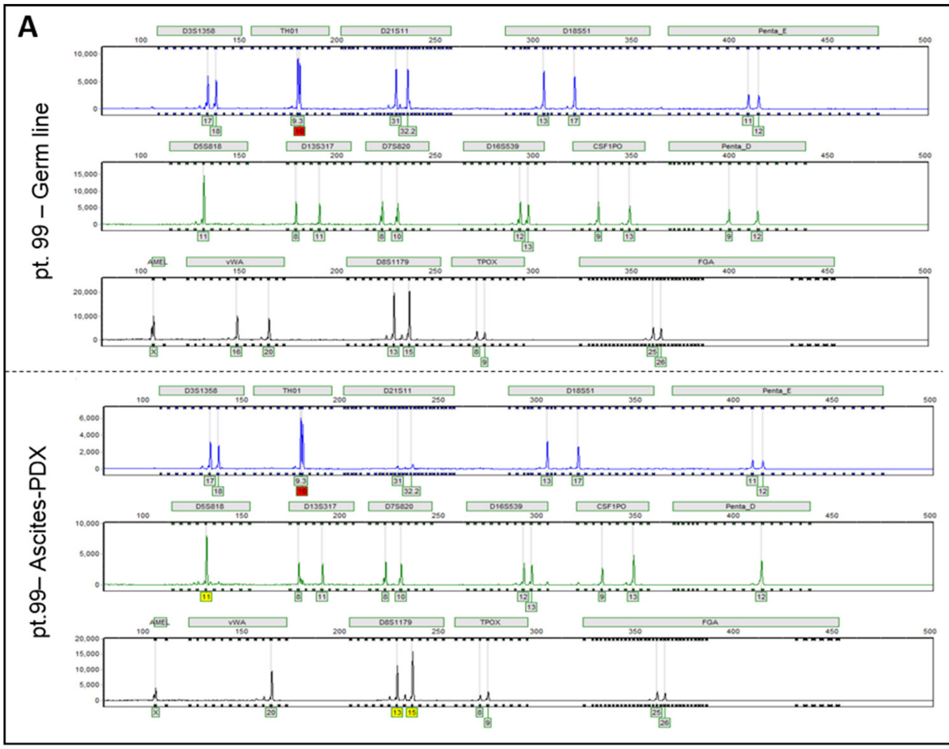

Pt ID	Percent match (%)	Number of LOHs
73	98.04	1
86	94.55	3
87	87.28	7
90	79.1	5*
99	96.55	2
105	86.27	7
116	1	0
144	86.27	3

LOH- Loss of heterozygosity
* Compared to ascites pellet

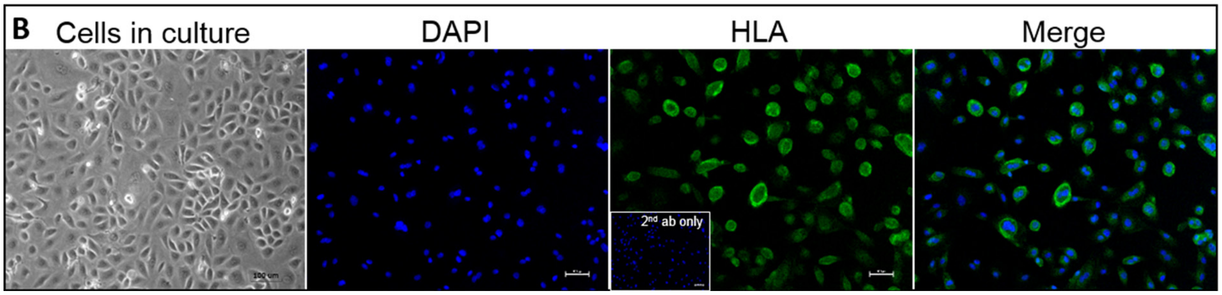

Supplementary Figure 1: STR authentication and cytological characterization of fluids. (**A**) PDX tumor authentication – single tandem repeats of germ-line (upper panel) and PDX tumor (lower panel) of 16 loci. Table – summary of loss of heterozygosity (LOH) and identity percentage. (**B**) Photomicrograph of representative ascites culture (left; scale bar-100 µm), and immunofluorescence cyto-staining with nuclear DAPI (second), positive HLA-A staining (third) (inset - merged of 2nd ab only stained cells), and merged photos (right). Magnification ×10, scale bar–100 µm.

Supplementary Table 1: Clinical characterisitcs and response to therapy

Patient ID	Gender	Stage at diagnosis	GL BRCA status	Treatment &response	Time from paracenthesis to exitus (days)	OS (months)	Established PDX
**As_#00073**	Female	IV	WT	1st line: Folfirinox; SD<br>2nd line: Gemcitabine+Abraxane; SD<br>3rd line: Gemcitabine+Oxaliplatin; PD	52	11	Yes
**As_#00086**	Male	IV	WT	1st line: Gemcitabine + SBRT; SD	31	3	Yes
**PE_#00087**	Male	IV	WT	1st line: Gemcitabine + Tarceva; SD<br>2nd line: Capecitabine; PD<br>3rd line: Folfox; PD	10	17	Yes
**As_#00090**	Male	IV	WT	1st line: Folfox; PD	34	1	Yes
**As_#00098**	Male	IV	WT	1st line: Folfirinox; SD<br>2nd line: Gemcitabine+Abraxane; SD<br>3rd line: Gemcitabine+ anti-angiogenic agent; NA	52	10	No
**As_#00099**	Female	IV	WT	1st line: Folfirinox; PR<br>2nd Immunotherapy; PD	49	13	Yes
**As_#00101**	Male	IV	WT	1st line: Folfirinox; SD<br>2nd line: SBRT; PD<br>3rd line: Gemcitabine; NA	58	10	No
**As_#00103**	Male	IV	NA	1st line: Folfirinox; SD<br>2nd line: Gemcitabine; NA	22	8	No
**As_#00105**	Male	III	WT	1st line: Folfirinox + SBRT; SD<br>2nd line: Gemcitabine+Abraxane; PD	72	7	Yes
**As_#00113**	Male	IV	NA	1st line: Folfox; NA	26	1	No
**As_#00116**	Female	IV	WT	1st line: Folfirinox; SD	49	6	Yes
**As_#00118**	Female	IV	WT	1st line: Folfirinox; PD	153	5	No
**As_#00144**	Male	II	WT	1st line: Folfirinox + SBRT; SD<br>2nd line: Capecitabine; PD<br>3rd line: Gemcitabine+Abraxane; NA	9	15	Yes
**As_#00135**	Male	IV	BRCA2 6174delT	1st line: Folfirinox; PD	14	4	Yes
**As_#00191**	Female	IV	NA	1st line: Folfirinox; NA	32	1	Yes
**As_#00194**	Male	II	WT	Adjuvant : Gemcitabine; NED<br>1st line: Folfox; NA	23	17	Yes
**As_#00195**	Male	III	WT	1st line: Folfirinox + SBRT; SD<br>2nd line: Gemcitabine+Abraxane; SD<br>3rd line: Immunotherapy; NA	27	11	Yes

Abbreviations:
GL- Germline; WT- wild type.
"SD - Stable disease; PR - Partial response; PD - Progressive disease; NED - No evidence of disease; ".
SBRT - Stereotactic body radiation therapy.
NA - Not applicable.

Supplementary Table 2: Common alterated genes in PDXs

Gene	Total count
KRAS	6
TP53	5
CDKN2A	4
FHIT	4
MIR4315-1	4
MIR4315-2	4
SRGAP2D	3
NRG1	3
CSMD1	2
FAM72C	2
CDH15	2
CTNNA2	2
DDR2	2
FOCAD	2
HS3ST4	2
IFNA16	2
IL1RAPL1	2
KIAA0195	2
LRRC7	2
MACROD2	2
MIR31HG	2
MIR6511B-1	2
MLLT3	2
NBEA	2
NRG3	2
NRXN2	2
PDE4D	2
PTPRD	2
SASH1	2
SMURF2	2
TGFBR2	2
TPTE	2
WWOX	2

Supplementary Table 3: Non-synonymous mutations in primary biopsy and PDX

Gene	Location	Coding/Amino Acid change	Variant Effect	dbSNP
PIK3CA	chr3:178952085	c.3140A > G/p.His1047Arg	Missense	rs121913279
KRAS	chr12:25380275	c.183A > C/p.Gln61His	Missense	rs17851045
FLCN	chr17:17118594	c.1337G > A/ p.Arg446His	Missense	
